# 3M™ Petrifilm Yeast and Mold Count Plate for the Enumeration of Yeasts and Molds in Dried Cannabis Flower: AOAC *Official Method*^SM^ 997.02

**DOI:** 10.1093/jaoacint/qsac114

**Published:** 2022-09-28

**Authors:** April Schumacher, Cari Lingle, Karen M Silbernagel

**Affiliations:** 3M Food Safety Department, 3M Center, Bldg. 260-6B-01, St. Paul, MN 55144-1000, USA; 3M Food Safety Department, 3M Center, Bldg. 260-6B-01, St. Paul, MN 55144-1000, USA; 3M Food Safety Department, 3M Center, Bldg. 260-6B-01, St. Paul, MN 55144-1000, USA

## Abstract

**Background:**

The 3M™ Petrifilm™ Yeast and Mold (YM) Count Plate is a sample-ready culture medium system that contains nutrients supplemented with antibiotics, a cold-water-soluble gelling agent, and an indicator system that facilitates yeast and mold enumeration.

**Objective:**

The 3M Petrifilm YM Plate was validated for enumeration of yeast and mold in dried cannabis flower through the AOAC Emergency Response Validation process.

**Methods:**

The performance of the 3M Petrifilm YM Plate was compared to that of Dichloran Rose Bengal Chloramphenicol (DRBC) agar. Matrix data were normalized by log_10_ transformation, and performance indicators included repeatability, difference of means, and inclusivity/exclusivity.

**Results:**

These studies demonstrated that the 3M Petrifilm YM Plate method detects and enumerates yeasts and molds from cannabis flower at low, medium, and high contamination levels, and the average log counts at 20–25°C for 5 days were equivalent to the average log counts of the DRBC reference method. In strain studies, 59 out of 60 yeasts and molds produced typical colony morphology on 3M Petrifilm YM Plates. Of the nontarget bacterial strains tested, 38 out of 39 strains were not detected on 3M Petrifilm YM Plates.

**Conclusions:**

The 3M Petrifilm YM Plate is a reliable method for the enumeration of live yeast and mold in dried cannabis flower.

**Highlights:**

The 3M Petrifilm YM Plate allows for detection of yeast and mold within 5 days of incubation. The sample-ready plates can be incubated at 20–25°C and can be stacked up to 20 plates, thus providing user flexibility and saving incubator space.

Yeast and mold are widespread in nature, can be found in the air, water, soil, and vegetation, and can grow in a wide range of environmental conditions. Because cannabis plants are grown in both outdoor and indoor conditions, plants grown outdoors are exposed to wider ranges and larger populations of fungal species. However, improper watering, type of soil and fertilizer, and poor air circulation can increase the chance of mold growth in indoor environments. Human handling during harvest increases the risk of secondary contamination for both indoor and outdoor-grown cannabis. The final product could develop fungi, or their growth byproduct if humidity and temperature levels of drying and curing rooms are not carefully controlled. Total yeast and mold count (TYMC) is used as an indicator of the overall cleanliness of the product’s life cycle, from growing, processing, handling, and to storage. Product with high TYMC can be detrimental to both consumers and cultivators.

While the majority of mold and yeast present in the environment are harmless, certain fungi cause spoilage and produce mycotoxins, a byproduct that is toxic to humans and animals. Several yeast and mold have been found to be prevalent in cannabis, including *Cryptococcus, Mucor*, *Aspergillus fumigatus*, *Aspergillus niger*, and *Aspergillus flavus* (1, 2). *Aspergillus* species *niger*, *flavus*, and *fumigatus* are known for aflatoxin production, a type of dangerous mycotoxin that can be lethal (3). For this reason, regulations exist to limit the allowable TYMC counts for purposes of protecting consumer safety (4).

The 3M Petrifilm Yeast and Mold Count (YM) Plate is a sample-ready culture medium system that contains nutrients supplemented with antibiotics, a cold-water-soluble gelling agent, and an indicator system that facilitates yeast and mold enumeration (5).

The 3M Petrifilm YM Plate was previously shown to be comparable to the US Food and Drug Administration *Bacteriological Analytical Manual* (BAM) Chapter 18 reference method (6) for enumeration of yeasts and molds in foods [58 food types, naturally contaminated and laboratory spiked (pre-collaborative study, data not published)] and has Final Action status as *Official Methods of Analysis*^SM^**997.02** (7). The current matrix extension study compares the performance of the 3M Petrifilm YM Plate to Dichloran Rose Bengal Chloramphenicol (DRBC) agar for the enumeration of yeasts and molds in dried cannabis flower [9-tetrahydrocannabinol (THC) >0.3%].

## Matrix Extension Validation Study

This validation study was conducted as an Emergency Response Validation (ERV) process within the AOAC Research Institute *Performance Tested Method*^SM^ program. The validation followed the *AOAC INTERNATIONAL Methods Committee Guidelines for Validation of Microbiological Methods for Food and Environmental Surfaces* (8) and the *Standard Method Performance Requirements (SMPR) for Viable Total Yeast and Mold Count Enumeration* (9), which was developed by the AOAC Cannabis Analytical Science Program. Inclusivity and exclusivity testing was conducted by 3M (St. Paul, MN 55144-1000) and Q Laboratories (Cincinnati, OH). Cannabis flower matrix study materials were prepared by Steadfast Analytical Laboratory (Hazel Park, MI). Test portions were blind-coded and provided to North Coast Testing Laboratories of Michigan (Adrian, MI) for analysis using the 3M Petrifilm YM Plate.

### Inclusivity/Exclusivity Study

An inclusivity/exclusivity study of the 3M Petrifilm YM Plate was previously performed using 31 strains of yeasts and molds and 40 strains of nontarget organisms in a pre-collaborative study (Q Laboratories, 3M data not published) as part of the AOAC *Official Methods of Analysis* **997.02** method validation (5). The ERV protocol required data for specific inclusivity and exclusivity organisms commonly found in cannabis matrixes. Additional strains to satisfy this requirement (29 inclusivity, 19 exclusivity) were tested by Q Laboratories.

Yeast organisms were propagated from a stock culture stored at –70°C to Potato Dextrose Broth and incubated at temperatures optimal for growth. Following incubation, yeast organisms were diluted to 100× the LOD of the 3M Petrifilm YM Plates. Mold organisms were propagated from a stock culture stored at –70°C to Sabouraud Dextrose Agar and incubated for 5–7 days at 30 ± 1°C. Following incubation, mold spores were harvested for inclusivity testing by washing cultures with Butterfield’s Phosphate Buffered Dilution Water. The mold wash was then diluted to 100× the LOD of the 3M Petrifilm YM Plates.

Exclusivity organisms were propagated from a stock culture stored at −70°C to trypticase soy agar with 5% sheep blood (SBA) and incubated at conditions optimal for growth. Following incubation, exclusivity organisms were transferred to the nonselective Brain Heart Infusion broth and incubated at conditions optimal for growth. Exclusivity cultures were analyzed undiluted.

All organisms were randomized, blind-coded, and plated onto 3M Petrifilm YM Plates as indicated in the instructions for use. Plates were incubated at 20–25°C and examined after 3 and 5 days. Final results were recorded after 5 days. Colonies were determined to be positive or negative based on the product instructions.

**Table 1.** Inclusivity results for the 3M Petrifilm YM Count Plates

No.	Organism	Source	Origin	Results^a^
1	*Alternaria alternata*	ATCC^b^ 13963	Not available	+
2	*Altemaria carthami*	ATCC 36748	Safflower seed	+
3	*Altemaria chlamydospora*	ATCC 28045	Desert soil	+
4	*Arthrinium* species (aureum)^c^	ATCC 56042	Not available	+
5	*Aspergillus aculeatus* ^c^	ATCC 56925	Grape	+
6	*Aspergillus brasiliensis*	ATCC 16404	Blueberry	+
7	*Aspergillus caesiellus* ^c^	ATCC 42693	Dried Chilies	+
8	*Aspergillus candidus*	ATCC 1002	Not available	+
9	*Aspergillus flavus*	ATCC 6943	Shoe Sole	+
10	*Aspergillus fumigatus* ^c^	QL^d^ 021116.3	Flour Tortilla	+
11	*Aspergillus nidulans*	ATCC 10074	Not available	+
12	*Aspergillus niger* ^c^	ATCC 6275	Not available	+
13	*Aspergillus ochraceus*	ATCC 1008	Not available	+
14	*Aspergillus oryzae* ^c^	ATCC 10124	Not available	+
15	*Aspergillus terreus* ^c^	ATCC 1012	Soil	+
16	*Aspergillus versicolor*	ATCC 9577	Human lesion	+
17	*Aspergillus vitricola*	ATCC 14567	Binocular lens	−^e^
18	*Aureobasidium* species (*pullulans*)^c^	ATCC 15233	Painted wood	+
19	*Botrytis cinerea*	ATCC 11542	Not available	+
20	*Byssochlamys fulva* ^c^	ATCC 24474	Canned Grape Juice	+
21	*Byssochlamys spectabilis* (formerly *Paecilomyces variotii*)^c^	ATCC 1114	Leather	+
22	*Candida albicans*	ATCC 10231	Man with Bronchymycosis	+
23	*Candida glabrata*	ATCC 2001	Feces	+
24	*Candida stellata*	ATCC 52826	Wild grapes	+
25	*Candida tropicalis*	ATCC 13803	Tea	+
26	*Candida tropicalis*	ATCC 13803	Not available	+
27	*Chaetomium globosum* ^c^	ATCC 6205	Stored Cotton	+
28	*Cladosporium pseudocladosporioides* (formerly *herbarum*)^c^	ATCC 58927	Air Sample	+
29	*Cladosporium allicinum*	ATCC 28987	Onion	+
30	*Cryptococcus neoformans* ^c^	ATCC 14116	Pigeon Nest	+
31	*Curvularia pseudobrachyspora* (formerly *lunata*)^c^	ATCC 12017	Tarpaulin	+
32	*Debaryomyces hansenii* ^c^	ATCC 60978	Cheese and Milk	+
33	*Dekkera bruxellensis* ^c^	ATCC 200341	Kombucha	+
34	*Fusarium oxysporum*	ATCC 48112	Not available	+
35	*Fusarium proliferatum* ^c^	QL 0567112-1C	Environmental isolate	+
36	*Fusarium solani* ^c^	QL 345317-4B	Environmental isolate	+
37	*Geotrichum candidum*	ATCC 34614	Clotted carrot	+
38	*Geotrichum silvicola* ^c^	QL 14282-1A	Milk	+
39	*Kluyveromyces spp*	Unknown^f^	Not available	+
40	*Mucor hiemalis* ^c^	ATCC 34334	Cow dung	+
41	*Mucor racemosus*	ATCC 46130	Not available	+
42	*Paecilomyces* species (*marquandii*)^c^	ATCC 10525	Soil	+
43	*Papiliotrema* (formerly *Cryptococcus*) *laurentii*^c^	ATCC 18803	Palm Wine	+
44	*Penicillium aurantiogriseum*	ATCC 60567	Not available	+
45	*Penicillium citrinum*	ATCC 36277	Not available	+
46	*Penicillium griseofulvum*	ATCC 11885	Not available	+
47	*Penicillium islandicum*	ATCC 26535	Wheat flour	+
48	*Phytophthora infestans* ^c^	ATCC MYA-1113	Potato tuber	+
49	*Pichia fermentans*	ATCC 10651	Buttermilk	+
50	*Purpureocillium* species (*lilacinum*)^c^	ATCC 10114	Soil	+
51	*Rhizopus oryzae* ^c^	ATCC 9363	Soy sauce	+
52	*Rhizopus stolonifer*	ATCC 14038	Not available	+
53	*Rhodotorula spp.*	Unknown	Not available	+
54	*Saccharomyces cerevisiae*	ATCC 38618	Not available	+
55	*Scopulariopsis acremonium* ^c^	ATCC 58636	Chicken house soil	+
56	*Stenella araguata*	ATCC 24788	Not available	+
57	*Talaromyces flavus* ^c^	ATCC MYA288	Microsclerotia of *Verticillium dahliae*	+
58	*Talaromyces pinophilus (Penicillium pinophilum)* ^c^	NRRL^g^ 11797	Corn	+
59	*Yarrowia lipolytica* ^c^	ATCC 9773	Not available	+
60	*Zygosaccharomyces rouxii*	ATCC 28253	Processed prunes	+

a The “+” symbol indicates typical colony morphology observed on Petrifilm YM Plates, and the “-” symbol indicates no growth observed on Petrifilm YM Plates.

b ATCC = American Type Culture Collection, Manassas, VA.

^c^ Strain tested by Q Laboratories to satisfy SMPR 2021.009 requirements. All other strains were tested as part of AOAC OMA **997.02**.

d QL = Q Laboratories Culture Collection, Cincinnati, OH.

e Per the ATCC strain information for *Aspergillus vitricola* ATCC 14567, this strain does not grow on common mold media such as Potato Dextrose Agar or Malt Extract Agar and requires supplementation of a growth medium with 70% sucrose.

f Unknown = Strain was tested in the pre-collaborative study, and the source identification was not included.

g NRRL = Agricultural Research Service Culture Collection, Peoria, IL.

**Table 2.** Exclusivity results for the 3M Petrifilm YM Count Plates

No.	Organism	Source	Origin	Results
1	*Acinetobacter baumannii* ^a^	ATCC^b^ 19606	Urine	–^c^
2	*Aeromonas hydrophila* ^a^	ATCC 49140	Clinical Isolate	–
3	*Bacillus cereus*	Unknown^d^	Not Available	–
4	*Bacillus stearothermophilus*	Unknown	Not Available	–
5	*Bacillus subtllis*	ATCC 6460	Not Available	–
6	*Bacillus thuringiensis*	Unknown	Not Available	–
7	*Burkholderia cepacia* ^a^	ATCC 25416	Plant Derived	–
8	*Citrobacter freundii*	Unknown	Not Available	–
9	*Clostridium perfringens*	Unknown	Not Available	–
10	*Edwardsiella tarda* ^a^	ATCC 15947	Human Feces	–
11	*Enterobacter aerogenes* ^a^	ATCC 13048	Sputum	–
12	*Enterobacter aerogenes*	Unknown	Not Available	–
13	*Erwinia amylovora* ^a^	ATCC 51852	Plant	–
14	*Escherichia coli*	Unknown	Not Available	–
15	*Escherichia coli O157: H7*	Unknown	Not Available	–
16	*Escherichia hermannii* ^a^	ATCC 33650	Mouse Brain	–
17	*Flavobacterium species*	Unknown	Not Available	–
18	*Hafnia alvei* ^a^	ATCC 51815	Milk	–
19	*Klebsiella oxytoca* ^a^	ATCC 43165	Clinical Isolate	–
20	*Klebsiella pneumonia* ^a^	ATCC 11296	Not Available	–
21	*Kluyvera species*	Unknown	Not Available	–
22	*Lactobacillus plantarium*	Unknown	Not Available	–
23	*Lactobacillus species*	Unknown	Not Available	–
24	*Listeria monocytogenes* ^a^	ATCC 7644	Human Isolate	–
25	*Micrococcus species*	Unknown	Not Available	–
26	*Morganella morganii* ^a^	ATCC 25829	Human	+^e^
27	*Pantoea agglomerans* ^a^	ATCC 19552	Sewage	–
28	*Proteus mirabilis* ^a^	ATCC 7002	Urine	–
29	*Pseudomonas aeruginosa* ^a^	ATCC 27853	Clinical Isolate	–
30	*Pseudomonas cepacia*	Unknown	Not Available	–
31	*Pseudomonas fluorescens* ^a^	QL^f^ 17041.3	Raw Milk	–
32	*Pseudomonas species*	Unknown	Not Available	–
33	*Rahnella aquatilis* ^a^	ATCC 55046	Soil	–
34	*Ralstonia pickettii* ^a^	ATCC 27511	Clinical Isolate	–
35	*Salmonella* Sandiego	Unknown	Not Available	–
36	*Salmonella* Senftenberg	Unknown	Not Available	–
37	*Staphylococcus aureus*	Unknown	Not Available	–
38	*Stenotrophomonas maltophilia* ^a^	ATCC 13637	Patient with mouth cancer	–
39	*Streptococcus faecalis*	Unknown	Not Available	–

a Strain tested by Q Laboratories to satisfy SMPR 009 requirements.

b ATCC = American Type Culture Collection, Manassas, VA.

c – = No growth observed on Petrifilm Yeast and Mold Count Plates.

^d^ Unknown = strain was tested in the pre-collaborative study, and the source identification was not included (Q laboratories, 3M data not published).

e 15 colonies of an approximately 10^8^ cfu/mL application of *Morganella morganii* grew on 3M Petrifilm YM Plates. Growth was also observed on DRBC media.

f QL = Q Laboratories Culture Collection, Cincinnati, OH.

### Matrix Study

Cannabis test materials were prepared by Steadfast Analytical from an inventory of retained samples from its Michigan licensed grower, patient, and caregiver customers. Samples were combined to produce batch materials of a low level (<1000 cfu/g), a medium level (1000–10 000 cfu/g), and a high level (10 000–100 000 cfu/g). Batches were manually mixed in an aseptic manner until homogeneous. For each contamination level, five replicate test portions (10 g) were quantified by spread-plating aliquots of diluted test portions onto DRBC agar plates. Table 3 summarizes the average cfu/g of yeast and mold for each contamination level that was provided to laboratories for analysis in the study.

**Table 3.** Average contamination level of yeast and mold in test batches

Batch	n^a^	DRBC^b^ (cfu^c^/g)
Low	5	350
Medium	5	5600
High	5	48 000

a n = Number of replicates.

b DRBC = Dichloran Rose Bengal Chloramphenicol agar.

c cfu = Colony-forming units per gram of cannabis material tested.

Individual 10 g test portions from each contamination level were placed in sterile filter Whirl-Pak bags. Five bagged test portions from each of the three contamination levels were selected for each candidate method participating in the ERV project. Test portions were assigned an identification tag in Michigan’s Marijuana Regulatory Agency seed-to-sale system for distribution and tracking. This served to blind-code the contamination level of the test portions. The test portions were also assigned random sample numbers for reporting results to AOAC.

Personnel from each of the participating independent laboratories were responsible for picking up and transporting the test portions to their laboratories on Monday, December 7, 2020. Participating laboratories were instructed to analyze samples on Tuesday, December 8, 2020 following the user guides provided with the candidate methods. In addition to the candidate methods, all test portions were enumerated using DRBC agar as described in DRBC Reference section. North Coast Testing Laboratories of Michigan conducted the matrix evaluation for the 3M Petrifilm YM Plate method.

### Candidate Method

All analyses were performed using paired test portions. Test portions were prepared for analysis as described in the 3M Petrifilm YM Plate method. Ten-gram portions were homogenized in 90 mL 0.1% peptone water (PW). Tenfold dilutions were made by transferring 10 mL into 90 mL PW and shaking 25 times in a one-foot arc within 7 s to ensure homogeneity. A 1 mL aliquot from each dilution (10^−1^, 10^−2^, 10^−3^, and 10^−4^) was plated in duplicate on 3M Petrifilm YM Plates. Plates were examined at 3 and 5 days, and final colonies were recorded at 5 days. Plates containing counts between 10 and 150 were used to determine the final results. If mainly yeasts are present, plates with 150 colonies are usually countable. When substantial amounts of mold were present and a more accurate count was obtained on the next dilution, the upper countable limit was lowered per the guidance in the 3M Petrifilm YM Plate method and BAM Chapter 18.

### DRBC Reference Media

Paired test portions, prepared following the candidate method dilution protocol, were confirmed by spread-plating aliquots of each dilution onto DRBC agar plates per the recommendation in the SMPR (9). From the initial dilution of the sample, 10 g (homogenized in 90 mL PW) 1.0 mL was spread-plated across two DRBC agar plates (0.5 mL on each plate) in triplicate (six total DRBC agar plates). Additionally, 0.1 mL of the 10^−1^, 10^−2^, and 10^−3^ dilutions was plated in triplicate on DRBC to obtain the 10^−2^, 10^−3^, and 10^−4^ dilutions, respectively. The agar plates were allowed to dry and were then incubated at 25 ± 1°C for 5–7 days before enumeration. Mold appeared as flat or fuzzy, spreading colonies with the natural pigmentation of the sporing structures, and yeast appeared as pink, smooth, raised colonies on DRBC agar plates (10). Plates containing counts between 10 and 150 colonies were enumerated as described in BAM Ch. 18.

        **AOAC Official Method 997.02**

**  Yeast and Mold Counts in Foods and Dried Cannabis Flower**

**    Dry Rehydratable Film Method (Petrifilm™ Method)**

**           First Action 1997**

**           Final Action 2000**

**Revised First Action 2021 (for Cannabis Flower, THC >0.3**%**, Only)**

[Applicable to enumeration of total yeasts and molds in foods and dried cannabis flower (THC >0.3%).]

*See* Tables **997.02A** and **B** for results of the interlaboratory study supporting acceptance of the method.

### A. Principle

The method uses culture plates of dry medium supplemented with antibiotics, dye to enhance visualization of growth, and cold-water-soluble gelling agent. Undiluted or diluted suspensions are added to plates at a rate of 1 mL/plate. Suspension is spread over a 30 cm^2^ growth area. Gelling agent is allowed to solidify, plates are incubated, and yeasts and molds are counted.

### B. Apparatus and Reagent

- *Yeast and mold count plates*.—Contain nutrients supplemented with chlortetracycline, chloramphenicol, cold-water-soluble gelling agent, and dye sensitive to presence of phosphatase (5-bromo-4-chloro-3-indolyl phosphate) that enhances visualization of yeast and mold growth. The circular growth area of a single plate contains 30 1 × 1 cm squares outlined on a film base (available as 3M™ Petrifilm™ Yeast and Mold Count Plates from 3M Food Safety, St. Paul, MN—Cat. No. 6407/6417).
- *Plastic spreader*.—Provided with Petrifilm plates, designed to spread suspension evenly over plate growth area.
- *Pipets*.—Serological pipet or pipetting syringe accurately delivering 1.0 mL.
- *Colony counter*.—Standard apparatus, Quebec model preferred, or one providing equivalent magnification (1.5×) and visibility.
- *Blender*.—High-speed mechanical blender rotating at 10 000–12 000 rpm, or stomacher.
- *Sterile diluents*.—Butterfield’s phosphate-buffered dilution water or 0.1% peptone water (PW).

### C. General Instructions

Store unopened 3M Petrifilm YM Plate pouches refrigerated or frozen at temperatures ≤8°C (46°F). Just prior to use, allow unopened pouches to come to room temperature before opening. Return unused 3M Petrifilm YM Plates to pouch. Seal by folding the end of the pouch over and applying adhesive tape. To prevent exposure to moisture, do not refrigerate opened pouches. Store resealed pouches in a cool, dry place for no longer than four weeks. It is recommended that resealed pouches of 3M Petrifilm YM Plates be stored in a freezer (see product instructions) if the laboratory temperature exceeds 25°C (77°F) and/or the laboratory is located in a region where the relative humidity exceeds 50% (with the exception of air-conditioned premises).

After use, plates contain viable yeast and/or mold cultures. Autoclave used plates 15 min at 121°C prior to discarding.

### D. Preparation of Test Suspension

Aseptically prepare 1:10 or greater dilution of food samples with sterile diluent. Blend or stomach 2 min and plate. Prepare additional dilutions as required. For dried cannabis flower (THC >0.3%), weigh out 10 g of sample from test portion into a sterile stomacher bag and dilute with 90 mL sterile 0.1% PW. Shake 25 times to homogenize. Prepare additional dilutions as required.

### E. Analysis

Place Petrifilm Yeast and Mold Count Plate on a flat surface. Lift the top film, hold the pipet perpendicular to plate, and carefully inoculate 1 mL test suspension onto the center of the film base. Place top film down onto inoculum.

Lift the plastic spreader using the circular handle. Align the center of the spreader with the approximate center of the plate. Distribute suspension evenly using gentle downward pressure on the center of the spreader. Do not slide the spreader across the film. Remove the spreader and leave the plate undisturbed 1 min to let the gel solidify.

Place the plates in the incubator in a horizontal position, clear side up, in stacks not exceeding 20 units. Incubate the plates 5 days at 20–25°C.

Count the plates promptly after incubation period. Yeasts appear as blue-green or off-white in color and form small defined colonies. Mold colonies are usually blue but may also assume their natural pigmentation (e.g., black, yellow, green). They tend to be larger and more diffuse than yeast colonies.

To calculate yeast and mold count, multiply total number of yeast and mold colonies/plate (or average number of colonies/plate, if counting duplicate plates of same dilution) by the appropriate dilution factor. When counting colonies on duplicate plates of consecutive dilutions, calculate the mean number of colonies for each dilution before determining average yeast and mold count.

Estimated counts can be made on plates with >150 colonies and should be reported as estimated counts. In making such counts, determine average count/1 cm^2^ and multiply by 30 (circular growth area is 30 cm^2^).

High numbers of yeast colonies may cause the entire growth area to turn blue. High numbers of mold colonies may cause growth area to turn blue, black, yellow, etc. When this occurs, do not make estimated counts, but further dilute and plate test suspension to obtain a more accurate count.

## Results

### Inclusivity/Exclusivity

Results from the inclusivity and exclusivity testing conducted at Q Laboratories have been combined with the results from the original 3M Petrifilm YM Plate AOAC *Official Methods of Analysis* validation and are presented in Table 1 (inclusivity) and Table 2 (exclusivity). All inclusivity yeasts and molds tested with the exception of *Aspergillus vitricola* American Type Culture Collection (ATCC^®^) 14557 (Manassas, VA) showed typical colony morphology and thus were considered “positive” on 3M Petrifilm YM Plates. *Aspergillus vitricola* ATCC 14557 is an osmophilic organism that did not recover on Potato Dextrose Agar as well as the 3M Petrifilm YM Plate. This is associated with the growth characteristics of this organism, which requires a very high glucose concentration. Thirty-eight out of the 39 exclusivity strains were not detected on the 3M Petrifilm YM Plates. One exclusivity strain, *Morganella morganii* ATCC 25829, had partial breakthrough growth on 3M Petrifilm YM Plates (15 colonies) and breakthrough growth on DRBC agar.

### Matrix Study

Paired statistical analysis was conducted for each contamination level comparing the candidate method result to cfu/g obtained on the DRBC agar plates. For each test portion, results were logarithmically (log_10_) transformed using the equation cfu/g + 0.1, according to the Least Cost Formulations, Ltd (2020) *Paired Method Analysis for Micro Testing* version 1.2 (Virginia Beach, VA). After transformation, replicate test portion results for each contamination level for each method were averaged, and the difference of means between methods with standard error and 95% and 90% confidence intervals were determined. Repeatability was also calculated for each contamination level for each method. The matrix study data are presented in Table 4.

**Table 4.** Matrix study: 3M Petrifilm YM Plate versus DRBC—Difference of means

Matrix	Cont. level	3M Petrifilm YM Plate		DRBC		DOM^d^	SE^e^	90% CI^a^		95% CI	
		Mean^b^	s_r_^c^	Mean	s_r_			LCL^f^	UCL^g^	LCL	UCL
Cannabis flower	Low	3.466	0.105	3.688	0.218	−0.221	0.073	−0.377	−0.065	−0.425	−0.018
	Med	3.861	0.045	3.906	0.047	−0.045	0.027	−0.103	0.013	−0.120	0.030
	High	5.270	0.107	5.145	0.204	0.126	0.048	0.023	0.228	−0.008	0.259

a CI = confidence interval for DOM.

b Mean of five replicate portions, after logarithmic transformation: log_10_[cfu/g + (0.1)f] where f is the smallest reportable result.

c s_r_ = Repeatability standard deviation.

d DOM = Difference of means.

e SE = Standard error of the mean difference for paired analysis.

f LCL = Lower confidence limit for DOM.

g UCL = Upper confidence limit for DOM.

**Table 997.02A.** Interlaboratory study results for determination of mold count in foods by dry rehydratable film method

Product	Mold level	Method	Mean log_10_ colony count	s_r_	s_R_	RSD_r_, %	RSD_R_, %	*r* ^a^	R^b^
Orange juice	Low	PYM^c^	2.50	0.13	0.17	5.05	6.93	0.36	0.49
		BAM^d^	2.50	0.33	0.38	13.23	15.17	0.94	1.07
	High	PYM	3.23	0.18	0.37	5.68	11.51	0.52	1.05
		BAM	3.21	0.12	0.36	3.66	11.10	0.33	1.01
Hot dog	Low	PYM	2.35	0.32	0.80	13.67	34.00	0.91	2.26
		BAM	2.20	0.08	0.98	3.44	44.69	0.21	2.78
	High	PYM	3.09	0.11	0.97	3.58	31.54	0.31	2.76
		BAM	3.06	0.19	0.98	6.18	31.89	0.54	2.76
Yogurt	Low	PYM	2.34	0.16	0.75	6.90	31.81	0.46	2.11
		BAM	2.15	0.11	0.92	5.18	42.68	0.31	2.59
	High	PYM	3.21	0.43	0.50	13.45	15.70	1.22	1.42
		BAM	3.00	0.17	0.92	5.50	30.49	0.47	2.59
Ketchup	Low	PYM	2.17	2.52	2.61	116.00	120.10	7.13	7.38
		BAM	1.90	0.27	0.67	13.93	35.08	0.75	1.88
	High	PYM	2.76	0.42	0.50	15.35	18.01	1.20	1.41
		BAM	2.78	0.18	0.80	6.32	30.50	0.50	2.40
Corn meal	Low	PYM	2.28	0.69	0.76	30.18	33.53	1.95	2.16
		BAM	2.29	0.39	0.63	17.13	27.56	1.11	1.78
	High	PYM	2.50	0.61	0.76	24.54	29.81	1.73	2.11
		BAM	2.54	0.51	0.64	20.01	25.10	1.44	1.80
Cake mix	Low	PYM	1.73	0.30	0.68	17.29	39.06	0.85	1.92
		BAM	1.57	0.52	0.82	33.35	52.29	1.48	2.31
	High	PYM	1.73	0.49	0.78	28.13	44.96	1.37	2.20
		BAM	1.71	0.31	0.77	18.26	45.01	0.88	2.18

a r = 2.8 × s_r_.

b R = 2.8 × s_R_.

c PYM = Petrifilm yeast and mold count plate.

d BAM = Method described in FDA *Bacteriological Analytical Manual,* 7th Ed., AOAC INTERNATIONAL, Gaithersburg, MD, USA.

**Table 997.02B.** Interlaboratory study results for determination of yeast count in foods by dry rehydratable film method

Product	Yeast level	Method	Mean log_10_ colony count	s_r_	s_R_	RSD_r_, %	RSD_R_, %	*r* ^a^	R^b^
Orange juice	Low	PYM^c^	1.72	0.48	0.77	28.05	44.98	1.36	2.18
		BAM^d^	1.72	0.51	0.82	29.41	47.81	1.43	2.33
	High	PYM	2.93	0.26	0.38	8.98	13.07	0.74	1.08
		BAM	2.95	0.15	0.36	5.20	12.32	0.43	1.03
Corn meal	Low	PYM	1.32	0.98	1.48	73.99	112.00	2.76	4.18
		BAM	1.49	0.81	1.45	54.00	96.88	2.28	4.09
	High	PYM	1.99	1.16	1.51	58.33	75.70	3.28	4.26
		BAM	2.27	1.08	1.32	47.85	58.15	3.07	3.73
Cake mix	Low	PYM	1.51	0.58	1.11	38.47	73.61	1.64	3.14
		BAM	1.23	0.75	1.18	61.10	96.59	2.12	3.35
	High	PYM	2.09	0.25	1.06	11.79	50.78	0.70	3.00
		BAM	2.07	0.40	1.14	19.25	55.01	1.13	3.22

a r = 2.8 × s_r_.

b R = 2.8 × s_R_.

c PYM = Petrifilm yeast and mold count plate.

d BAM = Method described in FDA *Bacteriological Analytical Manual,* 7th Ed., AOAC INTERNATIONAL, Gaithersburg, MD, USA.

## Discussion

The 3M Petrifilm YM Count Plate method was incubated at 20–25°C and compared to AOAC SMPR for Viable TYMC Enumeration at 5 days for the detection and enumeration of yeast and mold from dried cannabis flower. Naturally contaminated dried cannabis flower samples were tested at low, medium, and high levels. The log counts from the 3M Petrifilm YM Count Plate method were compared with log counts from DRBC agar.

The 90% and 95% confidence intervals indicated there were no significant differences in detection or enumeration between the 3M Petrifilm YM Count Plate method and the DRBC agar for dried cannabis flower samples at the low, medium, and high contamination levels.

In the inclusivity strain studies, 59 out of 60 yeast and mold strains were detected and had appropriate colony morphology on 3M Petrifilm YM Plates. In the exclusivity strain studies, 38 out of 39 nontarget strains tested were not detected on 3M Petrifilm YM Count Plate.

## Conclusions

These studies have demonstrated that the 3M Petrifilm YM Count Plate method is an accurate, specific, and repeatable method that detects and enumerates yeast and mold in 5 days at 20–25°C from dried cannabis flower. It is recommended that the 3M Petrifilm YM Plate, AOAC **997.02**, be granted a matrix extension for the detection and enumeration of yeasts and molds in dried cannabis flower.

## Conflict of Interest

3M Company is the method developer. All authors from 3M Company are salaried employees of the company. North Coast Testing Laboratories and Q Laboratories were contracted as independent laboratories to conduct the study per AOAC guidelines and received payment from 3M Company.
